# Indents

**Published:** 1919-10

**Authors:** 


### INDENTS

☞ **Brief Articles of Technical or Scientific Value will receive notice and comment. Contributions invited.**

The Education of a Dentist. One does not need to look far to discover the trouble in dental education. The whole fault lies in the organization of a separate training for dentists. The dentist should receive, in a large measure, the same training as the physician, and dentistry will never be a satisfactory component of medicine until the dentist and physician are on an educational parity. To bring about the necessary change in dental education, it will require several years and must be done by evolutionary rather than devolutionary methods. We face a condition, and not a theory, in the premises. The people must be cared for in the matter of dentistry, and with the increasing knowledge on the part of the layman of the dangers of mouth infections, there is going to be a corresponding increased demand for dental services. To meet this demand more dentists will be needed. It would seem, therefore, that the first responsibility in dental education is not to decrease the output of dentists, but strengthen the dental curriculum to a point which will bring about a change of character in the dental students. We have at present a four-year curriculum with a high school graduation for admission, and in 1921 the better dental colleges of the country will require one year of college for admission to the dental curriculum. This will necessarily decrease the output of dentists somewhat, and the requirement of one year of college for admission will probably have to stand for a number of years, at least, before any additional steps can be taken with safety. As soon, therefore, as the supply or demand will warrant, two years of college should be required for admission to the dental curriculum, which will place the dental student and the medical student on a basis of equality. When this step has been reached the first two years in dentistry and medicine may be identical. At the end of this period the dental student may undertake special training, and the medical student may also select his own special field of practice. A degree in medicine should then be conferred on both groups. We have before us an exceedingly difficult task, which must be faced with considerable thought and care. The supply must not be reduced seriously, and yet at the same time the training of the dentist must be of a different character if he is to functionate as an intelligent part of the medical program. It may be true that the question is too large for any early solution, and that we shall have to arrange for the special training of persons who will practice dentistry from a medical standpoint purely, and continue to train students for the general practice of dentistry as in the past.—Dr. F. B. Morehead, in *Journal A. M. A.*

---

MOUTH WASH FOR ORAL SUPPURATIVE CONDITIONS. Take Benzoic Acid, 2 drams; Thymol, 10 grains; Oil of Cassia, and Oil of Gautheria, of each 5 drops; Menthol, 10 grains; Boric Acid, 1 dram; Dilute Alcohol, (50%), 5 ounces; Distilled Water to make 16 ounces. Sweeten to taste and used in acceptable dilution.—Dr. Beecher, in *Cosmos.*

---

ASEPSIS IN FILLING ROOT CANALS. To my mind, a so-called complete root canal filling is the ideal result of an operation, the adjective "ideal" characterizing it as something that can not be accomplished. The only means we have at the present time to judge a root canal filling *in vivo* is by means of the X-ray, and everyone knows that such an examination will not permit the operator to state that the apical foramen and all the accessory foramina are hermetically sealed, nor that all the infection had been removed, nor that reinfection will not take place. The fact that a negative culture was obtained from a root canal just previous to its being filled does not guarantee that reinfection can not take place. I have yet to see the dentist who, during a root canal operation, will not forget himself at some time, and touch some object which will cause the infection of the field of operation. And I really can not blame him for it, because it certainly seems quite impossible to keep everything sterile during such a prolonged and intricate procedure. Even a root amputation is not advisable in case the patient is suffering from a systemic malady, as the remaining part of the root—no matter how treated—may act as a foreign body, and present a *locus minoris resistentiæ.*—Theodor Blum, *Dental Forum.*

---

The Dental Hygienist. Our helper is the dental hygienist. She is the channel through which the knowledge we have acquired of prevention can be disseminated. Our efforts must be analogous to those of the medical profession in regard to tuberculosis. We have no specific, but we do know means by which the disease can be prevented and controlled. What hope could the medical men have to prevent or control tuberculosis without the aid of the medical nurse? What hope have we to educate the public in the preventive methods that we know of except through the dental hygienist? The prophylactic treatment itself is not her greatest service. She is not a mechanical operator. The greatest service she can perform is the slow and painstaking education and enlightenment of the public in mouth hygiene and allied branches of hygiene. Primarily she must be a hygienist and educator; secondarily, a prophylactic operator.

A country-wide education and training of these women should be begun at once. The dental profession must accept this obligation to the public, and every state in the Union must make provision for the places of training. Fortunately it does not require a graduate dentist to enlighten the public in the prevention of dental diseases, although it is essential that he be able to do it. What it

*does* require is thousands of dental hygienists, women especially educated and trained, to care for the school children, and other thousands for service in private offices, orphanages, hospitals, and any other place where the dental profession attempts to fulfil its duty to humanity.—A. C. Fones, *Dental Items of Interest.*

---

THE STRASSBURG SCHOOL CLINIC. The famous clinic established by Dr. Ernest Jessen in Strassburg, Germany, was closed when the war took possession of that city, and when the French took the city of Strassburg Dr. Jessen fled to Basel, Switzerland, where he hopes to start a clinic for the school children of that city. Dr. Jessen started the first clinic of this sort in Strassburg in 1887, which he conducted from his own resources. In 1892 the city put the clinic on a secure financial basis and in 1902 gave it a well-appointed and equipped building. Up to the time of the war there were 220 school clinics in Germany. In some of the cities the service was gratuitous to the children and in most of them they paid a small fee of one mark annually. It is confidently expected that when peaceful relations are re-established, that the Strassburg clinic will be reopened, we hope with Dr. Jessen at the head of it. His great interest in this cause and his capacity to carry it on will make his services valuable in Switzerland, should he not be able to resume his work in Strassburg.

---

LOCAL ANESTHESIA IN CHILDREN. For some years Farr has been testing the application of novocain in surgical work on children. The operations performed have included those upon the head, neck, limbs, external genitals, spine and abdomen. In all 77 cases have been attempted with local anesthesia, and in only 7 of these has it been necessary to add inhalation anesthesia. Of these 77 cases, there was but 1 cleft-palate operation, on a child of fifteen years; and 4 cases of dissection of the neck for removal of enlarged glands, ranging in age from four to fifteen years. From this experience Farr draws the following conclusions:

(1) The psychic element is not as important in children as in adults when operating under local anesthesia.

(2) Less restraint is necessary during the administration of the local than during administration of general anesthesia.

(3) Much more tact and a more refined technique are required in operating upon children under local than under general anesthesia.

(4) The margin of safety possessed by novocain over general anesthesia is as great in children as in adults.

(5) A large percentage of bad risks should have the benefit of this margin of safety.

(6) More extensive application of novocain in the surgery of children is indicated, and if a more common use of this drug obtained in this class of cases, the science of medicine as well as the art of surgery will be benefited. —Dr. R. E. Farr, in *Cosmos,* and *Interstate Medical Journal,* for February, 1919.

---

Dental Hygiene in Rochester. The Rochester Dental Dispensary has taken on dental hygiene work for some of the country schools in addition to the great work it is doing now for the 33,000 children of the city of Rochester.

Mr. George Eastman has recently added $250,000 to his large gift to the dispensary, making a total of one and one-half million dollars in all.

---

Oral Hygiene Inspection for New York. The Educational Department of New York State has appointed Dr. Wm. H. Leak dental inspector in the public schools. His work will be largely the visiting of colleges, normal schools and lecturing on oral hygiene to teachers, clubs and school societies. It is hoped that teachers will not only impart practical instruction to pupils, but that a constant interest in dental hygiene may be stimulated.

---

Massachusetts Dental Hygiene Work. The state has appropriated $30,000 for oral hygiene in the state for the present year. This sum is wholly inadequate, but it will serve to keep up the interest if wisely expended and may be the means of starting a new interest in some parts of the state where nothing is being done at present.

---

New York City. The Board of Alderman of New York have decided to add nine additional dentists and eighteen hygienists to the dental workers in that city. This is progress and will help some, but it is wholly inadequate for the enormous amount of work to be done in that great city.

---

Chewing Gum at the Front. Did chewing gum help win the war?

"I'll say so," replies the doughboy, with a grin. And he knows!

"It was a necessity. We couldn't supply enough of it," answers the overseas welfare worker.

Soldiers on the firing-line used chewing gum to ward off thirst when canteens were empty and water not available. Chewing gum relieved many a parched throat on long marches and fulfilled a vital need to the men in the heavy artillery, who were constantly surrounded by clouds of thick dust while on duty behind the big guns.

Because of the small size and slight weight of chewing gum, it fitted into the smallest carrying space. Happy indeed was the soldier boy who, on opening his Christmas package from home delivered to him on Christmas morning by the American Red Cross, found candy-coated gum or a juicy stick tucked in the corners. Even chewing gum from the little old corner shop back home was enough to make a fellow whoop for joy. As one chap expressed it, "We were always glad to get chewing gum, just to have something to chew. A fellow gets kinda nervous when he's waiting for the signal to shoot ahead, and you sure can work off some of your queer feelin's with a gob of gum."

---

RED CROSS DENTISTRY. Three hundred and twenty-six of Uncle Sam's fighters were treated in the Red Cross dental ambulance in France in a period of two months. This miniature dental office on wheels was in charge of U. S. Army dentists loaned to the Red Cross, who did everything from pulling teeth to performing the most delicate operations known to dental surgery. Before the Army could provide proper facilities for dental work on troops in France, this ambulance was a veritable "godsend," as many commanding officers at the front expressed it. The men were treated at any camp where troops were billeted and even up to the first-line trenches. Many men were treated under shell fire and returned to their duties cured of that troublesome tooth. The dental ambulance consists of a regular dental chair with fixtures mounted on a closed automobile chassis. The equipment was supplemented by American instruments.

Covering a period of two months this dental ambulance treated 326 soldiers at 471 sittings for 431 cases of dental caries, 72 dento-alveolar abscesses, 21 gingivites, 10 devitalized pulps, 4 impacted teeth, 3 pericementites, 6 pulpites, 74 salivary deposits, 54 amalgam fillings, 2 amalgam and oxyphosphates, 243 oxyphosphates, 2 gutta-perchas, 27 root canals, 10 crowns reset, 5 bridges reset, 193 teeth extracted and 1 arsenical necrosis. In addition to the dental work done by this ambulance, the Red Cross had a free dental clinic at Red Cross Hospital No. 1 at Neuilly, formerly the American Ambulance Hospital, and afterwards operated by the Red Cross and the U. S. Army. Here doughboys, poilus and Red Cross workers had their teeth cared for by army dentists.

---

A SERIOUS BUT LUDICROUS ACCIDENT. The suggestion that there could be any causative relationship between the loss of masticating organs and the presence of a dinner fork in the stomach appears, at first sight, to be so far-fetched as to verge on the ludicrous. The circumstances under which the strange situation of a fork was indirectly and not remotely related to the absence of teeth are explained in the following extracts from a case reported in the *Lancet*, February 22nd:

"The patient gave the following history. Having lost all her upper teeth, and having had no plate fitted, she was unable to masticate her food thoroughly. On Christmas Day she swallowed a portion of a giblet which had some difficulty in passing down the gullet. Later she vomited, during which the undigested meat 'stuck in her throat' and caused difficulty in breathing. Thereupon she took a small dinner-fork from the kitchen table and passed it down the throat, handle first. She dislodged the foreign body and removed the fork. The piece of meat, however, again stuck in the gullet lower down. On attempting the same maneuver a second time she passed the handle of the fork 'a long way down.' To her dismay the constrictor muscles gripped the fork, and she gradually lost her hold upon the prongs and it disappeared. The patient applied to the hospital for relief; her story was not readily believed, but it was decided to take an X-ray picture, and the radiogram showed a dinner-fork in the stomach with the handle towards the pyloric end and the prongs towards the cardia."

A gastrotomy was done through a 2-inch abdominal incision, the fork was found lying with the prong end in the stomach and the handle in the duodenum, and it was removed, prongs first, through a $\frac{3}{4}$-inch incision in the anterior wall of the stomach. The whole operation took twenty minutes, and the patient made a successful recovery.—*Dental Record.*

---

Are You Ready to Practice Modern Dentistry? A new day has dawned for dentistry. Everywhere you go you will find people talking about the effect of the teeth on the health. Physicians are doing a great deal for dentistry in making inquiries of the patients who go to them for treatment as to the condition of their teeth. Lay periodicals are calling attention to the necessity for the care of the teeth and the deleterious effects of oral sepsis upon the general health. What does this mean to you?

If you are ready, it means the greatest boost up the ladder into public esteem which the profession of dentistry has ever experienced. We have been slowly climbing this ladder, but now the dream of every far-seeing dentist who has been laboring for the betterment of dentistry seems about to be realized.

People are being restored to health by dental treatment. The public are hearing more about dentistry than they ever heard before and they are talking about their friends who have been relieved of bodily suffering at the hands of the dentist.

It is not a fancy, it is a fact ,and withal it is the greatest happening in the profession since dentistry became recognized as something more than tooth-pulling.

Are you ready to properly treat and advise with the patients who come to consult you? In the parlance of the day, "Can you deliver the goods"? If not, whose is the fault?

Our literature and our society meetings have been largely given over to the problems of focal infection for several years. Special courses of instruction in radiography, bacteriology, root canal filling and kindred subjects have been conducted, all with the purpose of making you ready. Are you there? Pretty soon it will be too late and the procession will have passed on. Your opportunity will have vanished. Better read some, study some, and attend society meetings some, and get ready to fulfill your whole duty.—*New Jersey Dental Journal.*

---

THERAPY OF BUCCAL CANCER. Connected with the subject of the therapy of buccal cancer are two great factors. The first is that of early diagnosis—a factor of paramount importance in any case, but in none more so than in cancer of the buccal cavity. There is little excuse for failure in this respect, yet it is of frequent occurrence. There still are physicians who profess a great "fear of the knife," and are willing to carry patients for weeks and months until the disease has made such progress that the use of a knife does, indeed, become a fearsome thing. There is a second group of men, intelligent and honest, who, because the patient gives a history of chancre or a positive blood Wassermann reaction, forget the possibility of cancer in the presence of an ulcerating sore on the tongue and lose precious time in a vain endeavor to cure the lesion with arsphenamin and mercury. The second factor to be considered is the exceedingly rich lymphatic supply of the mouth and neck. This is important for two reasons: first, the possibility of early, deep-seated metastases, and second, the difficulty which it adds to efficient use of the roentgen ray and radium. Every one is familiar with patients in whom clean excision of a cancer of the mouth associated with persistent postoperative raying of the neck by competent roentgenographers has nevertheless been followed by the early appearance of deep cervical metastases. Since the roentgen ray exerts its influence in limiting cancer metastases by causing proliferation of connective tissues and of the endothelial cells of lymphatics to an extent sufficient to obliterate the lymph channels and tissue spaces, and also by a direct inhibitory effect on the cancer cells, it is obvious that, to a large extent, the richer and the more deeply situated the lymphatic supply, the less effective will be treatment by the roentgen ray. This tendency of buccal cancer to cervical metastases has led to the ultraradical, so-called "block dissection" for its cure, which consists in an attempt to remove the cancer and the lymphatic bearing structures of the neck en masse. High morbidity and mortality from infection, and failure to eradicate the tumor by this method, have caused many surgeons to go to the other extreme and to content themselves with local excision with the actual cautery or some form of high frequency current, and ligation of the external carotid artery, thus attempting to minimize recurrence by starving the tumor, and such limitation of metastases in the neck as may be afforded by the roentgen ray. While this method avoids the danger of infection to a large extent, it also fails to cure. Much has been claimed for radium in the treatment of cancer of the tongue; but while its usefulness as an adjunct to other measures is generally admitted, its dependability when applied by present methods to any but the most superficial lesions is yet to be demonstrated. In the management of buccal cancer we are, therefore, brought face to face with the one great, outstanding fact in present-day cancer therapy, namely, the utter necessity of early diagnosis, without which invasion of the rich lymphatic field of the face and neck places an enormous handicap on curative treatment.—*Journal, A. M. A.*

---

Tragedies of the Profession. There are many cases where operations have resulted directly or indirectly in the death of the patient, but the deaths occurring in the cases of the operators themselves are, happily, not many, and the most of them might have been averted had reasonable care been taken at the time of their occurring.

About three years ago, in the city of Portland, Oregon, an operator was engaged in the work of opening and cleansing a putrescent root canal. He was using a Gates-Glidden drill, and like a thousand other men who do the same work day after day in their practice, left the drill in the handpiece dangling at the end of the engine arm. Reaching for an antiseptic in his case, he inadvertently raised his knee and struck the end of the drill with sufficient force to have it pierce his clothing, and enter his leg just above the knee. Impatiently he thrust it aside, little dreaming of the results impending. Three hours afterward he was in an agony of pain, and later was taken to the hospital, where he died within twenty hours from the time he was struck. The poison from a rattler's fang would have hardly worked more quickly.

Dr. M., of Reno, Nev., was suffering one winter's day from a severe cold in his head, which had broken out in a rash or abrasion on the edges of his nostril. Some time during the day's work he had been handling a patient suffering from virulent syphilis, and at the end of two weeks the unfortunate doctor was a victim to that dreadful scourge. Unfortunately, he was in a poor state of health at the time; in fact, he had sought the high altitude of Nevada on account of the condition of his lungs. His system could not stand the task of throwing off the two poisons, and Dr. M. died, horribly diseased. He had unwittingly introduced the germ of syphilis to the abrasion on his nose.

A new assistant, poorly versed in her duties, a busy practitioner, and another Gates-Glidden drill that had not been sterilized, were the cause of the death of another operator of Anaconda, Mont. It seems that the assistant, instead of sterilizing the instruments she had found on the operating table, had picked up a drill that had been in use, and had put it in the bur stand remaining on the table. Dr. S., reaching across the table to pick up an instrument, had pricked his hand with the point of the drill. Blood poisoning set in, and the loss of the arm first did not stem the course of the poison; he had to yield his life to its ravages.—Gray McClintock, *Dominion Dental Journal.*

